# Structural Mechanism of a Partial Glucocorticoid Receptor Agonist Functioning as a Mineralocorticoid Receptor Antagonist

**DOI:** 10.1063/4.0000887

**Published:** 2025-10-27

**Authors:** Xu Liu

**Affiliations:** Emory University, Atlanta, Georgia, USA

## Abstract

The Glucocorticoid Receptor (GR) and Mineralocorticoid Receptor (MR) are homologous ligand-activated transcription factors that regulate extensive downstream gene expression networks involved in metabolism, development, electrolyte homeostasis, and inflammation. Various GR agonists have been developed for their anti-inflammatory effects in treating diverse disorders. However, many GR agonists also act as MR agonists, leading to undesirable side effects such as increased cardiac fibrosis and cardiomyopathy. MR antagonists are typically developed to circumvent the defects caused by MR overactivation, providing cardiac-protective benefits.

Vamorolone is a recently FDA-approved drug that represents a most promising treatment by simultaneously suppressing inflammation and cardiomyopathy in Duchenne muscular dystrophy (DMD) patients. It acts on both GR and MR but avoids safety concerns of the corticosteroid class, such as prednisolone. We set to bridge the gap in knowledge of molecular mechanism of vamorolone. Our published structural and biochemical studies showed that vamorolone, which lacks the C11-OH group, acts as a partial, dissociative GR agonist. Notably, the absence of the hydrogen bond between Asn564 and C11-OH observed in the GR-vamorolone structure allosterically increases conformational dynamics of the AF-2 site, changes the coregulator binding and alters the gene expression profile to drive dissociation of efficacy from side effects during treatment. In sharp contrast to the conventional drug prednisolone acting as a strong agonist for both MR and GR, vamorolone is an MR antagonist. X-ray crystal structure of the MR-vamorolone complex reveals the lack of residue Asn770-mediated hydrogen bond, distinguishing it from prednisolone binding. Vamorolone has similar coregulator binding profile as a known MR antagonist eplerenone, suggesting that MR adopts a unique conformation characteristic of the antagonist binding mode. These integrated studies lay the foundation for understanding how subtle chemical changes in ligands drive differential effects on a genome-wide scale and provide molecular insights for the design of more potent GR agonist and MR antagonist for better anti-inflammation and cardiac protection.

